# Of Malformation

**Published:** 1840-01

**Authors:** 


*Singular case of Malformation.*

On the 20th March of this year (1839), at Schneidemuhl, a stout woman who had before borne five living and healthy children, was delivered of a remarkably stout female infant, presenting the following defective formation. The heart, and, under it, the stomach, both organs apparently separated by a partition, lie outside of the thoracic and abdominal cavities, in a sack of skin, nearly transparent. The protrusion is through a deficiency of the lower third of the sternum and upper part of the wall of the abdomen, as far as midway between the pit of the stomach and navel. The fissure is altogether 5½ inches long, and 2¼ inches broad, and is situate almost in the middle line of the body. The child is living, sucks, and is otherwise well. The prolapsed parts are protected by an appropriate bandage.—*Med. Zeitung. April* 17, 1839.

